# Research on the network handoff strategy based on the best access point name decision

**DOI:** 10.3389/fncom.2022.1090301

**Published:** 2023-01-10

**Authors:** Zhong Shu, Ya-Jie Shu, Qian Zhang, Hui-Rong Ye

**Affiliations:** School of Electronic and Information Engineering, Jingchu University of Technology, Jingmen, China

**Keywords:** Kalman-Bucy filtering, Lagrangian function, potential game, Jacobi matrix, network handover

## Abstract

To improve the network switching performance and efficiency of mobile phone terminals and establish an efficient mobile communication network connection, this paper constructs the SDN+MPTCP+CP (Software Defined Network, Multi-Path TCP, and Mobile Terminal) mobile communication network model and designs a network switching algorithm with a preselected available access point name (APN) based on the potential game method. The constructed network model integrates a 5G mobile communication network, satellite communication, the SDN network, and the MPTCP multi-way communication technology. APN access point is preselected by using Kalman filtering theory, and dual problems are resolved with the Lagrange function. To determine the MPTCP sub-flow transmission path, the differential derivative calculation is introduced. The performance of the network switching strategy is evaluated based on the Jacobian matrix. Then, the game coefficient is designed, and the game function is calculated. A potential game balance point is found, and an updating strategy is formulated to determine the best APN access point. The simulation network model is constructed, and the parameters of the performance evaluation are defined to find the performance comprehensively. The experimental results demonstrated the extreme reliability, stability, and compatibility of the proposed algorithm.

## 1. Introduction

In the heterogeneous wireless communication network, certain information-receiving devices, such as mobile satellite telephones, and onboard network components, are equipped with various network access ports to ensure the multipath transmission control protocol (MPTCP) (Qu et al., 2015; Teng et al., 2020) that can be applied to these devices. There are two main network communication modes: the communication between equipment, and the communication between equipment and the access network.

In the MPTCP communication mode, the mobile information-receiving device terminal transmits the collected image or video information through the network, leading to the network switching problem (Sinky et al., 2016; Tan et al., 2018; Yao et al., 2020; Tong et al., 2021). Network switching refers to the mobile communication network of mobile phones to realize network connection in real-time, due to mobile terminal devices. Network switching frequently occurs by increasing access point name (APN) deployment areas, thus degrading the data transmission quality.

The leading research on network handoff (Paasch et al., 2012) combines the application of the MPTCP communication protocol, defines the MPTCP data sub-flow as an advanced tool, and designs three data transmission strategies for all sub-flow, part sub-flow, and one independent sub-flow to judge whether vertical switching can be performed between Wi-Fi networks and 3G mobile networks. The more classical horizontal switching study (Croitoru et al., 2015) aims to introduce the MPTCP communication mode in the Wi-Fi network and obtain the best Wi-Fi access point through statistical calculations and analysis of the network congestion situation. With the development of data centers and Internet data services, MPTCP mobile communication networks have been studied in the literature (Williams et al., 2014; Mena et al., 2017), for example, by analyzing the performance of both MPTCP and TCP networks, taking the network throughput and network delay as the evaluation parameters, so the transmission performance of the MPTCP sub-flow has been verified in multiple transmission paths.

Moreover, the impact of mobile terminals on the transmission quality of sub-flow in high-speed motion has been verified by analyzing the transmission performance of sub-flow in multiple MPTCP networks. In the case of very frequent network switching, a particular mobile terminal network switching system was designed by Li et al. (2018). They found that the quality of the transmission performance of the MPTCP network is closely related to the network switching technology by evaluating the transmission performance of both TCP and MPTCP networks. In the case of accessing the satellite communication port, the MPTCP network model based on satellite communication was designed, the detection rules of MPTCP sub-flow in the transmission process, and the necessity of multi-route scheduling of the neutron traffic transmission path was demonstrated by Rene et al. (2015).

With the extensive applications of 5G communication networks (Luo et al., 2019) and the development of relevant research on 6G communication networks when the real-time monitoring and network data transmission of the running status of the Internet of vehicles and some high-speed vehicles are under consideration, some scholars have studied the deployment and application of 6G mobile communication networks (Saada et al., 2019; Zong et al., 2019; Gui et al., 2020). Relevant research illustrated the critical problems in applying 6G communication network technology and proposed some solutions for them (Zhang et al., 2018; Tang et al., 2019; Yang et al., 2019). To realize reliable MPTCP sub-flow multi-path transmission, the MPTCP communication network was integrated with the software-defined network (SDN) by utilizing the advantages of the SDN technology in centralized and expansible network management.

The manuscript researches the network handoff when the mobile terminal shows real-time changes in motion speed and the improvement of sub-flow transmission performance by the MPTCP. The SDN technology is employed to improve the network switching mode. Moreover, game theory is introduced to enhance the network throughput of the MPTCP sub-flow transmission, thus reducing the network transmission delay, and controlling the packet loss rate. The research focuses on vertical switching between mobile devices and the access network system to find the best APN access point and ensure stable network throughput in information transmission.

The main contributions of the research: constructing the SDN+MPTCP+CP model based on the fusion of SDN, MPTCP, and Mobile Terminal, designing the APN signal strength detection method, presenting the network switching method, introducing game theory to improve the network switching efficiency, and enhancing the network throughput. The evaluation parameters of network performance such as transmission delay and sub-stream packet loss rate are also integrated into the model.

The rest of the article is constructed as follows: Section 2 presents the network model. Section 3 presents the proposed algorithm. Section 4 presents the outcomes of conducted experiments. The research is concluded in Section 5.

## 2. Network model

**FIGURE 1:**
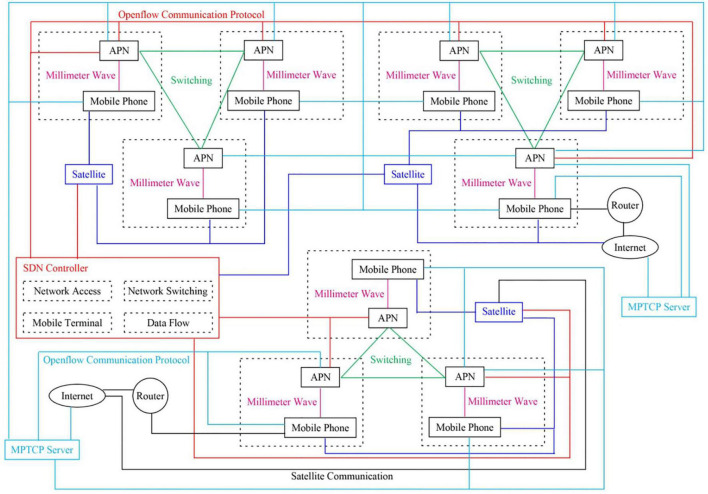
The mobile network communication model constructed in this manuscript.

The manuscript adopts the MPTCP communication mode to construct a mobile network model, mainly used to access mobile phone communication terminals and mobile phone networks. The constructed network model is called SDN + MPTCP + CP. Figure 1 shows the MPTCP mobile network model constructed based on the SDN technology.

Figure 1 depicts that the SDN controller can establish communication connections with all satellite transponders and all mobile network information forwarding units. Mobile phone users can communicate with satellite transponders and mobile network information forwarding units within the same area. Network switching is mainly utilized to select suitable satellite transmitters and mobile network forwarding units within different regions. the MPTCP server can establish communication connections with the Internet, a satellite transponder, and a router. The network model introduces the SDN technology with the SDN controller as the main component. The SDN controller includes four different control modules: network access, data flow control, network switch control, and mobile phone terminal control.

The controller is mainly employed to manage network resources, collect and analyze the status information of network operation, formulate MPTCP sub-flow transmission path scheduling, control mobile phone users to switch between different access networks, and ensure that mobile phone users can find the best and available access points. The main body of the mobile network comprises a satellite transponder, a mobile network information forwarding unit, and a mobile phone.

Although a 5G or 6G mobile network can be used, the mobile phone must provide a satellite signal reception interface. The transmission of data and information is completed through the MPTCP servers of the communication parties. The MPTCP server mainly collects the location or activity status information of mobile phone users and saves the relevant network operation status and network management information. The communication data includes the MPTCP server, connected Internet, and routers.

## 3. The implementation of the algorithm

### 3.1. Detection of the intensity of APN signal

To collect the location and activity information of mobile phone users, the communication connection should be first realized with mobile phone users. The APN mobile network information forwarding unit is the main device that realizes the connection with mobile phone users. The strength of the APN signal detected by mobile phones is the premise of establishing a communication connection between the two. The APN with high signal strength is the first choice of mobile phone users and the primary network handoff requirement. When a signal is detected, the mobile phone selects one with a high signal strength between two or more APNs to establish a communication connection, which can be implemented by using Kalman filtering. Some notations are presented as follows: the intensity value of the APN signal determined last time is denoted by A1, the actual detected APN signal intensity is denoted by A2 (in this case, A1 = A2), and A3 denotes the current determined APN signal intensity value. The error covariance of the APN signal intensity value determined last time is indicated by B1, the currently determined error covariance of the APN signal intensity value is defined as B2, the noise covariance generated during the previous and the current determination process is denoted by C1, where B1 = B2+C1 and the detected noise covariance caused by the determination error is denoted by C2. The correction coefficient of the Kalman filter is denoted by K,

where K = B1/(B1 + C2), A1 = A2+K × (A3-A2), and B2 = (1-K) × B1.

The value of A1 is the maximum detected signal intensity value, corresponding to the APN as the preferred wireless network access point for the mobile phone user.

### 3.2. Network handoff algorithm

The designed network handoff algorithm considers five factors related to network performance: throughput, consumption, switching efficiency, MPTCP sub-flow packet loss, and congestion. Specific requirements are as follows: (1) the total network throughput of all MPTCP sub-flow transmission paths in the network switching cannot exceed the throughput of any sub-flow transmission path before the network switching; (2) network bandwidth occupied by all MPTCP sub-flow transmission cannot exceed the amount of network bandwidth occupied by any sub-flow transmission path. To define the transport path of the MPTCP sub-flow, it is divided into ordinary and enhanced transmission paths, and the sub-flow transmission process can quickly switch between these two transmission paths.

The set of all mobile phone terminals is defined as *D*, where *D* = {1, 2,…,*d*}, and the number of all MPTCP sub-flow is denoted by E, where *E* = {1,2,…,*e*}. The *d*th mobile phone terminal can realize network transmission through the *e*th sub-flow with the following features: *E* = {*e, d, d*∈*D*}. The set of sub-flow transport paths is defined as *L*, where *L* = {1,2, …,*l*}. The collective capacity of *L* is denoted by *F(L)*, which is the sub-flow capacity provided by the *l*th sub-flow transmission path, and *f’(l)* is the actual sub-flow capacity of the *l*th sub-flow transmission path. *γ(l,e)* indicates whether the *e*th sub-flow is transmitted through the *l*th path, where *γ(l, e)* = 1 for positive values, and *γ(l, e)* = 0 for negative values. The transmission rate of *e*th sub-flow at a specific time t is denoted by *v(e, t)*, and *g(e, t)* is the network congestion, which is the amount of sub-flow information at time *t*. *τ(e)* is the transmission delay of the *e*th sub-flow, where *v(e, t)* = *g(e, t)*/τ*(e)*. The packet loss rate of the sub-flow is described by *h(e)*, where the packet loss rate of the transmission path is denoted by *h(l)*. The packet loss factors of the network switching are defined as α*(e)*, β*(e)*, and δ*(l)*, respectively, which are the positive gain coefficients for transmission sub-flow and transmission path. *V(e, t)* is the main control parameter of the proposed algorithm denoted by


$$v\,\left(e,t\right)=m\,\left(e,d\right)\times {\left(n\,\left(e\right)\times \beta \,\left(e\right)-\frac{h\,\left(e\right)\times \alpha \,\left(e\right)}{2}\right)}_{v}^{+}\,\,a\,n\,d$$

(1) $$\,h\,\left(l\right)=\delta \,\left(l\right)\times {\left(f^{\prime }\,\left(l\right)-f\,\left(l\right)\right)}_{h}^{+}$$

where *m(e, d)* represents the sub-flow information collected by the mobile phone terminal; *m* denotes the acquisition cycle; *n(e)* represents the network switching control parameter; *N*(*e, m*) denotes the network switching transfer factor, where $n\,\left(e\right)=N\,\left(e,m\right)\,/\,\sum_{e=1}^{E}N\,\left(e,m\right)$, *N*(*e*,*m*) = *A*1(*m*)/*ln*⁡(1 + τ(*e*,*m*)). The switch *A1(m)* denotes the APN signal intensity information collected in an acquisition period, and *τ(e, m)* represents the acquisition delay in an acquisition period. thus, the solution method of the proposed algorithm can be obtained as:


(2) $$\begin{array}{c} n\,\left(e\right)\times \beta \,\left(e\right)<\frac{h\,\left(e\right)\times \alpha \,\left(e\right)}{2}\Rightarrow v\,\left(e\right)=0\,a\,n\,d\\ v\,\left(e\right)>0\Rightarrow \beta \,\left(e\right)=\frac{h\,\left(e\right)\times \alpha \,\left(e\right)}{2\times n\,\left(e\right)}\\ f^{\prime }\,\left(l\right)<f\,\left(l\right)\Rightarrow h\,\left(l\right)=0\\ a\,n\,d\,h\,\left(l\right)>0\Rightarrow f^{\prime }\,\left(l\right)=f\,\left(l\right) \end{array}$$

To compute the effectiveness of *β(e)*, it is an essential guaranteed condition of the algorithm. *Q*(*d*,*v*) ∈ *E*^|*d*|^ denotes the validity solution function concerning the effectiveness of the set E, where *E*^|*d*|^ is a concave function.

Moreover, considering *f*′(*l*)≤*f*(*l*), the goal of the solution function is: $\max\,\sum_{d=1}^{D}Q\,\left(d,v\right)$, expressed as the maximum sub-flow transmission rate, and can also be expressed as minimizing network congestion. Taking *v*(*e, t*) as the primary calculation parameter, *L(v, h)* is the main calculation target according to the definition of the Lagrange function that is obtained according to the dual problem solution theory as:


(3) $$\begin{array}{c} L\,\left(v,h\right)=\sum_{d=1}^{D}Q\,\left(d,v\right)-\sum_{l=1}^{L}h\,\left(l\right)\times \left(f^{\prime }\,\left(l\right)-f\,\left(l\right)\right)=\\ \sum_{d=1}^{D}Q\left(d,v\right)-\sum_{l=1}^{L}h\left(l\right)\times \\ \left(\sum_{e=1}^{E}\gamma \,\left(l,e\right)\times v\,\left(e\right)-f\,\left(l\right)\right)\\ =\sum_{d=1}^{D}\left(Q\,\left(d,v\right)-\sum_{e=1}^{E}v\,\left(e\right)\times h\,\left(e\right)\times \beta \,\left(e\right)\right)+\\ \sum_{l}^{L}h\,\left(l\right)\times f\,\left(l\right) \end{array}$$

In Eq. 3, the extreme value is calculated using the partial derivative, and the Eq. 4 is attained,


(4) $$\begin{array}{c} \frac{\partial Q\,\left(d,v\right)}{\partial v\,\left(e\right)}<h\,\left(e\right)\times \beta \,\left(e\right)\Rightarrow v\,\left(e\right)=0\,a\,n\,d\\ v\,\left(e\right)>0\Rightarrow \frac{\partial Q\,\left(d,v\right)}{\partial v\,\left(e\right)}=h\,\left(e\right)\times \beta \,\left(e\right)\\ f^{\prime }\,\left(l\right)<f\,\left(l\right)\Rightarrow h\,\left(l\right)=0\\ a\,n\,d\,h\,\left(l\right)>0\Rightarrow f^{\prime }\,\left(l\right)=f\,\left(l\right)\\ \frac{\partial Q\,\left(d,v\right)}{\partial v\,\left(e\right)}=2\times n\,\left(e\right)\times \beta \,\left(e\right) \end{array}$$

The reliability, stability, and compatibility of the computations presented above can be verified according to the following conditions:

1. According to the definition and symmetry properties of the Jacobi matrix $\left(\frac{\partial Q\,\left(d,v\right)}{\partial v\,\left(e\right)}={\left[\frac{\partial Q\,\left(d,v\right)}{\partial v\,\left(e\right)}\right]}^{T}\right)$, the partial derivatives of *Q(d, v)* exist. This assumption is the fundamental premise of the above operations and is a reliable guarantee for the application of the calculation presented above. Meanwhile, the value ${\left[\frac{\partial Q\,\left(d,v\right)}{\partial v\,\left(e\right)}\right]}^{T}$ should be negative and continuous.
2. The relationship between the transmission flow and the transmission rate can be accurately described if $\frac{\partial f\,\left(l,d,h\right)}{\partial h\,\left(l\right)}\leq 0\,\,a\,n\,d\,\,\lim_{h\,\left(l\right)\to \infty }f\,\left(l,d,h\right)=0$, where $f\,\left(l,d,h\right)=\sum_{l=1,e=1}^{L,E}\left(\gamma \,\left(l,e\right)\times v\,\left(e\right)\times h\,\left(e\right)\right)$.
3. If $\lim_{h\,\left(l\right)\to \infty }\beta \,\left(e\right)=\infty$ denoting the sub-flow transmission rate is 0, there must be network congestion. Although the network congestion should be controlled, evaluation conditions are required to evaluate the severity of the network congestion state.
4. The sub-flow transmission has good compatibility if $\beta \,\left(e\right)\leq \frac{1}{{\left(v\,\left(e\right)\times \tau \,\left(e\right)\right)}^{2}}$.

The rapid implementation of network switching is crucial for determining *β(e)*. Suppose that the control factor defining the network handoff is denoted by *ε(e)*, then


(5) β⁢(e)=ε⁢(e)/v⁢(e).

The response speed of network handoff is mainly related to three parameters: *β(e), m(e, d)*, and $\frac{\partial Q\,\left(d,v\right)}{\partial v\,\left(e\right)}$, while the key parameter is $\frac{\partial Q\,\left(d,v\right)}{\partial v\,\left(e\right)}$, where $\frac{\partial Q\,\left(d,v\right)}{\partial v\,\left(e\right)}=-\frac{\varepsilon \,\left(e\right)}{v\,\left(e\right)}=-\frac{2}{{\left(\tau \,\left(e\right)\right)}^{2}\times {\left(v\,\left(e\right)\right)}^{3}}$.

The conditional equation based on the response speed of the TCP communication protocol in network handoff is higher than that of the MPTCP communication protocol. Accordingly, the following relation can be obtained:


$$\max{\left(\sum_{e=1,e\in d}^{E,E\in D}v\left(e\right)\right)}^{3}=\max\frac{2\times {\left(v\,\left(e\right)\right)}^{2}}{\varepsilon \,\left(e\right)\times {\left(\tau \,\left(e\right)\right)}^{2}}\Rightarrow$$

(6) $$\varepsilon \,\left(e\right)=\frac{2\times \max{\left(v\left(e\right)\right)}^{2}}{{\left(\tau \,\left(e\right)\right)}^{2}\times {\left(\sum_{e=1,e\in d}^{E,E\in D}v\,\left(e\right)\right)}^{3}}$$

By utilizing Eq. 5, the increment coefficient that defines the subsequent sub-flow transmission velocity *o(e)* is calculated by


(7) $$o\,\left(e\right)=n\,\left(e\right)\times \frac{2\times \max{\left(v\left(e\right)\right)}^{2}}{{\left(\tau \,\left(e\right)\right)}^{2}\times {\left(\sum_{e=1,e\in d}^{E,E\in D}v\,\left(e\right)\right)}^{3}}.$$

According to the above discussion, the main network handoff process aims to define a switching threshold W to judge the strength of the APN. When the signal strength value of the APN is higher than the threshold W, the network handoff method described in Eqs 2, 4, 6 are employed to generate the first common transmission path for sub-flow transmission. On the other hand, when the signal strength value of the APN detected by the mobile terminal is lower than the set threshold W, the network handoff method described by Eqs 4, 6 can be employed to generate two enhanced transmission paths for sub-flow transmission. The main codes of Algorithm 1 are as follows:

**Algorithm 1:** Network switching algorithm

**Input parameters:** SDN+MPTCP+CP, E, etc. **Output parameters:** Implement Algorithm 1 Initialize the parameters A1, v(e), τ(e), w, and β(e) **While** transferring all sub-streams **do** **if** A1 ≥ w **then** Establish a communication connection using the SDN+MPTCP+CP network model $n\,\left(e\right)=N\,\left(e,m\right)\,/\,\sum_{e=1}^{E}N\,\left(e,m\right)$ and *n*(*e*,*m*) = *A*1(*m*)/*ln*⁡(1 + τ(*e*,*m*)) β(e) = 1 Complete the operations between Eqs 2 through 6 Compute statistics A1, v(e), τ(e), w, β(e) Start a normal transmission path for sub-stream transmission **else** Perform the process A1 < W **end if** **if** A1 < w **then** Establish a communication connection by the model called SDN+MPTCP+CP β(e) = 0 Complete the operation between Eqs 2 through 6 Compute statistics A1, v(e), τ(e), w, β(e) **else** Perform the process A1 ≥ W **end if** Implement Algorithm 1 **end while**

### 3.3. APN selection algorithm

To further optimize the selection of APN access points, the manuscript introduces the game theory (Liang et al., 2021) and designs an optimization algorithm for mobile terminals to select the APN access points based on the potential game.

The set of all mobile phone terminals in the network is defined as *D*, and *D* = {1, 2,…,*d*}, the optional set of the APN access points for mobile terminals is denoted by *I*, and *I* = {*1*, 2, …, *i*}. The set of all network switching modes used by the *d*th mobile phone terminal is denoted by *X(d)*, where *X(d)* = {*x(1), x(2),…,x(d)*}. A certain network switching mode is represented by *x(d)*, when *x(d)* > 0, the mobile phone terminal d should switch over the network to establish a connection to the next APN; when *x(d)* = 0, the mobile phone terminal d maintains an existing network connection with the current APN. *A(d, i, t)* denotes the *ith* APN signal strength received by the *d*th mobile phone terminal at a certain time. *Y(d, I, t)* denotes the network bandwidth allocated by the *d*th mobile terminal to receive the sub-flow transmission with the *i*th APN at a certain time. *H(d, i, t)* represents the packet loss rate of the *d*th mobile terminal to receive the sub-stream transmission with the *i*th APN at a certain time. Then, the performance evaluation parameter *Z(d, i, t)* of all mobile terminals for network switching between the entire APNs can be determined. Moreover, the performance evaluation parameter of network switching can be determined for a specific current APN.

When multiple mobile phone terminals switch to the same APN access point, the APN access point may reduce the bandwidth allocated to the mobile phone terminals, leading to network congestion and packet loss in sub-flow transmission. To avoid this occurrence, a quality function *Z*(*t*) can be constructed for judgment.


(8) $$Z\,\left(t\right)=z\,\left(d\right)\times \mu \,\left(x\,\left(d\right),0\right)+\sum_{d=1}^{D}\sum_{i=1}^{I}\left(z\,\left(d,i\right)\times \mu \,\left(x\,\left(d\right),i\right)\right)$$

where μ(*x*(*d*)) denotes the performance evaluation factor. When *x*(*d*) = *i*, then μ(*x*(*d*)) = 1; otherwise, μ(*x*(*d*)) = 0.

The signal strength and network handoff mode should be considered while choosing the APN access point of the mobile phone terminal *x(no-d)* denote the network handoff mode of the mobile terminal except for that of the *d*th, where *x(no-d)* = {*x(1),x(2),…,x(d-1)*}, that is, the *x(d)* switching mode is not employed. Then, the *d*th mobile phone terminal could find the best network switching mode *x(d)*, and the best switching mode that is calculated can be defined as *x*(*yes*-*d*), *x*(*yes*-*d*) ∈ *arg*⁡*max*⁡(*Z*((*yes*-*d*),(*no*-*d*))), where ((*yes*−*d*),(*no*−*d*)) represents the combined value of the best switching mode.

All mobile phone terminals in set *D* are defined as individuals participating in the game, and all APN access points are regarded as game goals. According to the definition of the equilibrium point in the potential game theory, the value calculated by the game is the equilibrium point *x*(*yes*−*d*). In the combination of ((*yes*−*d*),(*no*−*d*)), when *Z*(*x*(*yes*−*d*),*x*(*no*−*d*))≥*Z*((*yes*−*d*),*x*(*no*−*d*)) is satisfied, it must exist *x*(*yes*−*d*). *y*(*m*−*apn*) denotes the network bandwidth between the *m*th phone terminal *d(m)* and the APN access points. *y*(*n*−*apn*) represents the network bandwidth between the *n*th phone terminal *d(n)* and the APN access point. Both *h(m)* and *h(n)* denote the packet loss rates of the *m*th and *n*th phone terminals, respectively. θ represents the potential game coefficient. If θ(*d*(*m*) = *d*(*n*)), *m*th and *n*th mobile terminals have selected a common APN access point. If θ(*d*(*m*) > 0), the *m*th terminal has at least one optional APN access point. θ(*d*(*m*) = 0) means that the *m*th phone terminal has not found the APN access point. The potential game function ω(*d*) can be described as:


(9) $$\begin{array}{c} \omega \left(d\right)=\frac{1}{2}\sum_{m=1}^{D}\sum_{n=1}^{D}(\frac{d\,\left(m\right)}{h\,\left(m\right)}\times y\left(m-apn\right)\times \frac{d\,\left(n\right)}{h\,\left(n\right)}\\ \times y\left(n-apn\right)\times \theta \left(d\left(m\right)=d\left(n\right)\right)\times \theta \left(d\left(m\right)>0\right)\\ +\sum_{m=1}^{D}\frac{d\,\left(m\right)}{h\,\left(m\right)}\times y\left(m-apn\right)\times \theta \left(d\left(m\right)=0\right) \end{array}$$

If the *d*th mobile phone terminal finds the next best APN access point, the original network switching mode (*yes*−*d*) will be changed $\left(y\,e\,s-\vec{d}\right)$. The performance evaluation parameters of the combined network handoff will have the relationship represented by $Z\,\left(\left(y\,e\,s-\vec{d}\right),x\,\left(n\,o-d\right)\right)>Z\,\left(\left(y\,e\,s-d\right),x\,\left(n\,o-d\right)\right)$, and the potential game function also has the relationship denoted by $\omega \,\left(\left(y\,e\,s-\vec{d}\right),x\,\left(n\,o-d\right)\right)>\omega \,\left(\left(y\,e\,s-d\right),x\,\left(n\,o-d\right)\right)$. According to the description of these two relationships and the definition of Eqs 8, 9 is attained,


(10) $$\begin{array}{c} \omega \left(\left(yes-\vec{d}\right),x\left(no-d\right)\right)-\omega )(\left(yes-d\right),x\left(no-d\right))\\ =\frac{d\,\left(i,t\right)}{h\,\left(i,t\right)}\times y\,\left(i,t\right)\,\sum_{m=1\,a\,n\,d\,m\,\neq \,i}^{D}\frac{d\,\left(m\right)}{h\,\left(m\right)}\\ \times y\left(m-apn\right)\times \theta \left(d\left(m\right)=\left(yes-\vec{d}\right)\right)\\ -\frac{d\,\left(i,t\right)}{h\,\left(i,t\right)}\times y\left(i,t\right)\sum_{m=1\,a\,n\,d\,m\,\neq \,i}^{D}\frac{d\,\left(m\right)}{h\,\left(m\right)}\times y\left(m-apn\right)\times \theta (d\left(m\right)\\ =\left(yes-d\right))\\ \omega \,\left(\left(y\,e\,s-\vec{d}\right),x\,\left(n\,o-d\right)\right)-\omega \,\left(\left(y\,e\,s-d\right),x\,\left(n\,o-d\right)\right)>0 \end{array}$$

According to the above discussion, the main code of the APN access point optimization algorithm based on the potential game is as follows:

**Algorithm 2:** Potential game APN access optimization algorithm.

**Input parameters:** D, A, Z, H, Y, etc. **Output parameters**:$\omega (\left(yes-\vec{d)},x\left(no-d\right)\right)$ ω((*yes*−*d*),*x*(*no*−*d*)) > 0, ω(*d*) d(I,t), φ(*x*(*d*)), *Z*(*t*), θ, (*no*−*d*), (*yes*−*d*), $\left(y\,e\,s-\vec{d}\right)$, ω(*d*) **while** *x*(*yes*−*d*) ∈ *arg*⁡*max*⁡(*Z*((*yes*−*d*),(*no*−*d*))) $\omega \,\left(\left(y\,e\,s-\vec{d}\right),x\,\left(n\,o-d\right)\right)>\omega \,\left(\left(y\,e\,s-d\right),x\,\left(n\,o-d\right)\right)$ **do** Compute statistics d(I,t) $Z\,\left(t\right)=z\,\left(d\right)\times \mu \,\left(x\,\left(d\right),0\right)+\sum_{d=1}^{D}\sum_{i=1}^{I}\left(z\,\left(d,i\right)\times \mu \,\left(x\,\left(d\right),i\right)\right)$ Calculate μ(*x*(*d*)) and *Z*(*t*) **if** $\omega \,\left(\left(y\,e\,s-\vec{d}\right),x\,\left(n\,o-d\right)\right)>\omega \,\left(\left(y\,e\,s-d\right),x\,\left(n\,o-d\right)\right)$ **then** Run Eq. 8 Compute statistics θ, (*no*−*d*), (*yes*−*d*), $\left(y\,e\,s-\vec{d}\right)$, ω(*d*) Select $x\,\left(y\,e\,s-\vec{d}\right)$ network switching mode **else** *x*(*no*−*d*) **end if** Send the best APN access point to the mobile terminal **end while**

**FIGURE 2:**
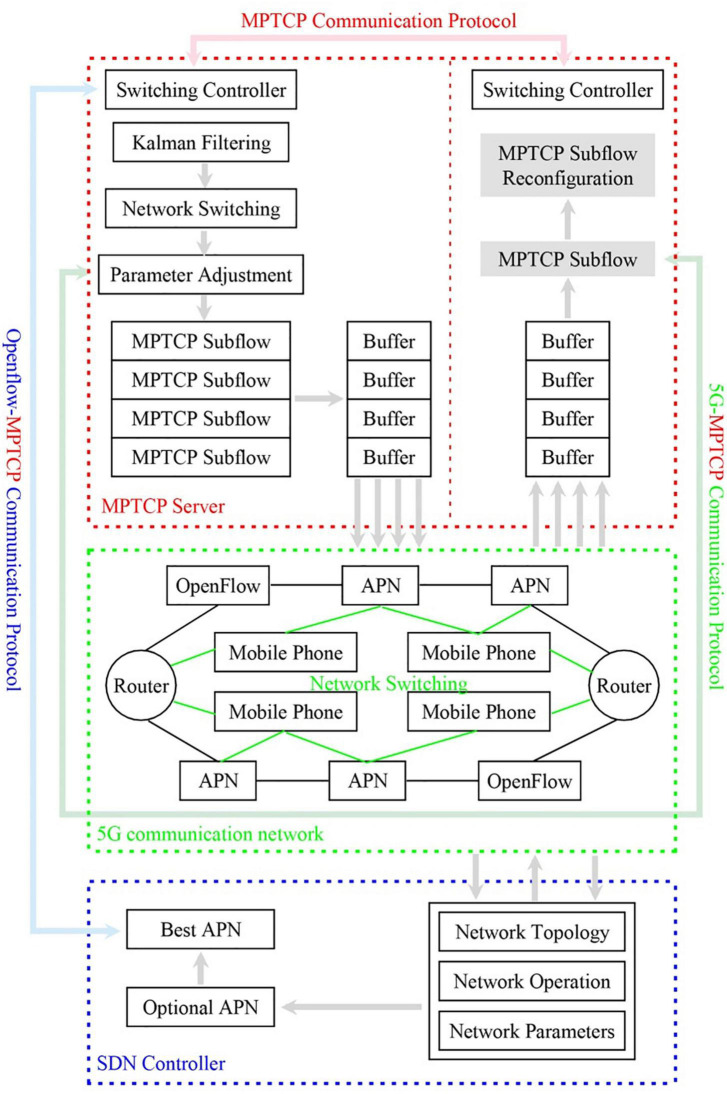
Flow diagram of the network switching information transmission based on the potential game.

Algorithm 2 can be run in Algorithm 1. Figure 2 shows the flow chart of data information transmission of the network switching algorithm based on the designed potential game.

The MPTCP Communication Protocol performs the data transmission of a communication. At the same time, the 5G-MPTCP Communication Protocol performs information transmission using 5G and MPTCP compatible communication protocols, and OpenFlow MPTCP Communication Protocol performs information transmission using OpenFlow and MPTCP compatible communication protocols.

## 4. Experimental results and discussion

### 4.1. Experimental environment and experimental parameters

Figure 1 depicts that the SDN+MPTCP+CP simulation network system was constructed that mainly employed the 5G communication network part presented in Figure 2 as the diagram of experimental network topology. The experiment has 20 tested mobile phone terminals, 10 APN access points, 2 OpenFlow switches, 2 satellite transponders, 2 MPTCP servers, and one SDN controller. The network bandwidth was set to 20 Mbps, and the total cache capacity in the MPTCP server module was 20M bp. The transmitted file information was the status file of the movement process of each mobile phone terminal, the movement time was 3,600 s, and the movement speed was randomly distributed to 0–3 m/s speed. Except for the movement environment of the mobile phone terminal specified by the experiment, the unspecified movement environment was a well-shaped environment designed by simulation. The movement environment area was 10,000 m^2^, the distance between the satellite transponder and the motion environment was set to 20,000,000 m, and the communication interface of all equipment was simulated on the mobile phone terminal, including the satellite transponder interface.

The experiment mainly verified the performance of the proposed algorithm, and the main contents were presented as follows:

(1) The basic performance of the SDN+MPTCP+CP network model was verified. The performance indicators of the experimental evaluation included: cache utilization B (%) in the MPTCP server module, the number of optimal APN access points N(*pcs*), mobile terminal network switching efficiency W (%), network delay T(*ms*), network bandwidth utilization P (%), MPTCP sub-flow packet loss rate K (%), and network throughput S (M/s).
N denotes the number of APN access points connected within the 3,600 s of mobile terminal movement. T represents the time for the mobile phone terminal to the MPTCP server to complete a communication transmission within 3,600 s, excluding 3,600 s. According to the total network bandwidth, the cache capacity is set to 20Mbp. The usage of a sub-flow transmission cache detected at a specific time in the sub-flow transmission is denoted by X1, and X2 denotes the sub-flow receiving cache usage, the bandwidth usage of the receiver MPTCP server detected at a specific time in the sub-flow transmission is denoted by X3. The number of 10 mobile terminals that realize network switching through the algorithm A1 at a certain time is described by Y1. The total number of mobile phone terminals participating in the experimental test is denoted by Y2. The number of all sub-flow of the MPTCP server during a communication transmission is represented by Z1. The total number of sub-flow of the transferred files is denoted by Z2. The MPTCP server transmission rate tested during a communication transmission process is represented by V1. The OpenFlow switch transmission rate is denoted by V2. The SDN controller transmission rate is described as V3. Thus,

$$B=\frac{X\,1+X\,2}{2\times 20},W=Y\,1\,/\,Y\,2,P=X\,3\,/\,20,K=Z\,1\,/\,Z\,2.$$

$$S=\frac{V\,1+V\,2+V\,3}{3\,T}$$

(2) The reliability, stability, and compatibility of the Algorithm 1 were verified. The performance indices of the experimental evaluation include four parameters: Jacobian matrix value [H], sub-flow transmission flow C(*Mbp*), network congestion evaluation parameter D, and sub-flow transmission compatibility parameter F.
[H]is the Jacobi matrix corresponding to the maximum value of the sub-flow transmission path calculated based on the extreme value of partial derivatives, and its value is negative and continuous. D means that the normal transmission rate of the sub-flow is nonzero, but a state with a value of 0 can be detected. F describes the positive gain coefficient of the transmission sub-flow, which was less than or equal to one, the sum of the sub-flow transmission rate and network delay is squared. The transmission volume of the MPTCP server tested during a communication transmission is denoted by X4. X5 denotes the OpenFlow switch transmission, and the SDN controller transmission is denoted by X6,
where $C=\frac{X\,4+X\,5+X\,6}{3}$.
(3) The performance of the ordinary path sub-flow transmission algorithm (abbreviated as A1) was compared with the enhanced path sub-flow transmission algorithm (abbreviated as A2) and enhanced path sub-flow transmission algorithm (abbreviated as A3) based on the potential game.
(4) The sub-flow transmission performance of Algorithm1 + Algorithm2 (abbreviated as A4) was evaluated when the mobile phone terminal was in a different motion environment. Also, according to the network configuration conditions and performance parameter evaluation method of Experiment 1, the mobile phone terminal motion environment included a linear motion environment (abbreviated as B1), a cross-motion environment (abbreviated as B2), and a well-shaped motion environment (abbreviated as B3). the presented algorithm in this experiment included the Algorithm1+Algorithm2 strategy and the APN signal intensity detection rule, abbreviated as A4.
(5) The performance of A4 was compared with Teng et al. (2020) (abbreviated as A5), (Yao et al., 2020) (abbreviated as A6), and (Meng et al., 2022) (abbreviated as A7) according to the conditions of a network configuration and the performance parameter evaluation method of Experiment 1. More references can be found in Ahmad et al. (2022), Alrashed et al. (2022), and Singamaneni et al. (2022).

### 4.2. Experimental results and discussion

According to the above network environment and experimental requirements, the experimental statistics are shown in Tables 1–5.

**TABLE 1:** Performance data of the network model.

Index parameters	B (%)	N (*pcs*)	W (%)	T (ms)	*P* (%)	K (%)	S (M/s)
Parameter values	93	17	98	65	86	4	16

**TABLE 2:** Performance data for Algorithm 1.

Index parameters	[H]	C (Mbp)	D	F
Parameter values	Yes	18	Yes	Yes

**TABLE 3:** Performance data for the four-seed flow transmission policy.

	Index parameters						
Algorithm	*B* (%)	*N* (pcs)	*W* (%)	*T* (ms)	*P* (%)	*K* (%)	*S* (M/s)
A1	54	83	59	193	47	32	2
A2	73	29	77	91	62	19	9
A2 + A1	84	21	89	75	78	11	12
A3	91	14	96	68	89	6	16

**TABLE 4:** Performance data for the three exercise environments.

	Index parameters						
Environment	*B* (%)	*N* (pcs)	*W* (%)	*T* (*ms*)	*P* (%)	*K* (%)	*S* (M/s)
B1	95	15	96	66	87	5	14
B2	92	14	94	69	86	6	17
B3	93	16	91	70	84	5	15

**TABLE 5:** Performance data for the four different algorithms.

	Index parameters						
Algorithm	*B* (%)	*N* (pcs)	*W* (%)	*T* (ms)	*P* (%)	*K* (%)	*S* (M/s)
A4	93	16	94	65	87	6	13
A5	89	23	87	73	72	9	8
A6	87	26	83	76	76	12	6
A7	91	19	89	68	81	10	10

The data in Table 1 evaluates the performance of the designed entire network model. Index parameters B focused on assessing the resource utilization of the MPTCP server module, index parameter N focused on assessing the network switching control effect of the SDN controller, index parameter W focused on assessing the 5D network between the mobile terminal and the network switching efficiency of the APN access point, index parameters T, P, K, and S were employed to comprehensively evaluate the performance of flow transmission, network switching and update, and network resource utilization in the whole network model.

The cache consumption in the MPTCP server module was slightly higher than that of the received amount when sending a sub-flow, indicating that the network switching process had little impact on the subsequent sub-flow transmission. Overall, the cache setting capacity was high, ensuring the efficient transmission of the sub-flow. In the SDN control module, the optional APN access points were far more than the number of the best APN access points, demonstrating the significant impact of the network switching strategy in the proposed algorithm.

In the 5G network module, network switching occurred among mobile phone terminals, APN access points, and satellite transponders. However, the network switching with the satellite transponder was less. So, the APN access points connected to the mobile phone terminals came from the best APN access point, and the position of the APN access points changed frequently. It further suggested that the network switching strategy designed by the proposed algorithm was fully applied to realize the autonomous update of network switching.

The actual network delay of experimental detection was higher than the movement time of 3,600 s of the mobile phone terminal, which was compatible with the actual state of the real environment. It indicated that the statistical T value in 1 was the value obtained by subtracting 3,600 s from the detected actual network delay, and the network delay of sub-flow transmission increased in the three main modules. The experiment detected that the network bandwidth usage of the MPTCP server sender, 5D network, SDN controller, and MPTCP server receiver was not much different. In contrast, the SDN controller and the MPTCP server receiver were slightly lower.

The packet loss was detected during the 3,600 s movement time of the mobile phone terminal. By enabling the network switching and update strategy, the reception of all streams can be ensured. The network delay of the lost bun flow was slightly 65 ms in Table 1, indicating that the lost bun flow enabled the multi-path transmission function in the new transmission, and the MPTCP communication rules were fully utilized.

The network throughput in Table 1 was calculated according to the minimum sub-flow transmission speed. At the sending terminal of the MPTCP server, the sub-flow transmission occupied all the network bandwidth, and the sub-flow transmission speed was equal to the network bandwidth value. The sub-flow transmission speed of the subsequent links indicated a downward trend, but the decrease was insignificant. So, the minimum value of the sub-flow transmission speed appeared at the receiving end of the MPTCP server. However, time was not enough to complete the packet loss sub-flow reception, indicating that the packet loss of the sub-flow transmission did not necessarily occur in the last period of 3,600 s.

The statistics in Table 1 indicated that the designed SDN + MPTCP + CP network model met the basic requirements of MPTCP, 5G, and OpenFlow communication rules, and these three communication rules had good compatibility. Meanwhile, it also demonstrated the significant impact of the network switching strategy based on the potential game algorithm.

The statistics in Table 2 were employed to verify the reliability, stability, and compatibility of Algorithm 1. The experiment evaluated the performance of the transmission path enhanced by sub-flow, and one of the essential intermediate parameters was the flow C of sub-flow transmission.

The value of [H] was expressed as the Jacobian matrix, and the ideal value of [H] included both negative and continuous values. The values calculated in Table 1 indicated that if the conditions of both aspects were satisfied, the positive value was taken as YES. The negative value is assigned to NO if any condition cannot be satisfied. The Jacobian matrix was used for the construction of a sub-flow transmission path rather than finding the construction of an ideal APN access point network switch directly since transmission paths could be constructed for APN access points. The Jacobian matrix can also include the mobile terminal and APN access point devices in the transmission path. However, it cannot fully describe the sub-flow on other related devices. Moreover, the sub-flow transmission path can fully represent the sub-flow transmission process in the whole network. More importantly, it can include the sub-flow transmission flow. In the experiment, the value of [H] was always negative, the value of each moment was different, and the test value was hung according to a specific decrease in the transmission process, indicating the strong reliability of Algorithm 1.

The C value of sub-flow transmission flow considered three main links: MPTCP server, OpenFlow switch, and SDN controller. The performance of the whole sub-flow transmission process was comprehensively evaluated. The statistics obtained from the experiment were 18 Mbp, which was close to the total bandwidth of 20 Mbp, and the lowest data detected in the three main links were 17 Mbp, indicating that the sub-flow was faster and in a better state.

The evaluation parameter of network congestion D suggests that any link in the sub-flow transmission process has sub-flow passing through. Therefore, the sub-flow transmission rate can be employed for direct evaluation. In the experiment, when the sub-flow transmission rate was greater than 0 under normal conditions, the positive value of D was YES; otherwise, the negative value of NO was taken. Within 3,600 s, the system detected no sub-flow transmission rate of 0 at any time, and the minimum value of the sub-flow transmission rate was 13 Mbps, indicating no network congestion in the sub-flow transmission process and implying the excellent stability of the network operation. The sub-flow transmission rate was equal to or less than 0, which indicated that it could be found while network congestion occurs.

The sub-flow transmission compatibility parameter F represented the sub-flow transmission performance among the four main communication protocols, namely, MPTCP, OpenFlow, 5G, and satellite during the sub-flow transmission, which was also a comprehensive evaluation of the performance of the whole network system. The value of F combined two factors: sub-flow transmission rate and network delay. According to the definition of a positive gain, the smaller the coefficient of a positive gain, the larger the product coefficient of the positive gain would be. Thus, any parameter value represents the transmission performance of the sub-flow.

Therefore, it is reasonable to reduce the value of *F*. When the experimentally set F value was less than or equal to one, the sum of the sub-flow transmission rate and the network delay is squared. The positive value of F was YES; otherwise, the negative value of NO was taken. The experiments indicated that while the *F* value was the smallest in the MPTCP sub-flow sending phase, the *F* value was the largest in the MPTCP sub-flow receiving phase. Thus, the value was also positive in the 5G and SND modules. The F value in Table 2 demonstrated that there was no network congestion at the interface of each link of sub-flow transmission. The information conversion among the four main communication protocols was assessed as normal, and the whole transmission path was called smooth.

The statistics in Table 3 could evaluate the performance of A1, A2, and A3. A1 employed the Kalman filter to calculate the APN access point, A2 strengthened the switch to calculate the APN access point, and A3 used the potential game to obtain the APN access point by strengthening the switch when the strongest signal value is under consideration.

In A1, APN access points were substantial. However, it was challenging to determine the final sub-flow transmission path selection without screening, thus causing the network switching efficiency, cache utilization, bandwidth utilization, network throughput, and network extension. The sub-flow packet loss rates were high. The experiments indicated that all the sub-flow could be received, and the sequence was disrupted in the received sub-flow. Besides, it demonstrated that using an ordinary path for sub-flow transmission was insufficient, and substantial improvements are required.

In A2, the performance evaluation indicators had been significantly improved without adding the rules of Algorithm 1, because the APN access points mainly had been screened. In the experiment, two strategies of using both Algorithms 1, 2 were added, and the value of the performance evaluation index was improved to some extent, indicating the importance of finding the best APN access point.

In A3, the APN access points were screened three times, and the obtained APN access points were found to be the best, which reduced the network delay and improved the transmission rate of sub-processes. The results of this experiment resemble those of the first experiment, indicating excellent stability of the presented algorithm.

The statistics in Table 4 evaluated the sub-flow transmission performance using the complete A4 algorithm in the manuscript when the different environments of the mobile phone terminal are utilized. The B1 environment was moving back and forth in a straight line, and the available APN access points were found to be the least. The B2 environment was moving back and forth on two straight lines, and a few more APN access points could be selected. The B3 environment was moving back and forth on four straight lines, and the maximum number of APN access points could be selected. Table 4 showed that the performance impact of sub-stream transmission was found to be insignificant for various numbers of APN access points to select the best APN access point. The fewer optional access points, the more opportunities to be repeatedly listed as the best access point would be. Thus, it would be easier to achieve rapid network switching.

Table 5 compares the sub-flow transmission performance of four different network switching algorithms. A4, A5, A6, and A7 were employed in the experiment by using the network configuration conditions and the evaluation methods of performance parameters in Experiment 1.

To resolve the problems of single data transmission mode and the significant influence of the environmental factors on training mobile communication networks, the A5 algorithm employed the SDN technology and network virtualization technology to evaluate the security and reliability improvement in the data transmission process by establishing a network model that integrates both space and earth. The SDN technology was mainly employed for the centralized management and virtual mapping of the bottom sources of the network. The problem can be resolved by establishing an association between the network’s bottom resources and the data transmission software. The common point with the A4 algorithm in the manuscript was the constructed network model. The problems of long network switching times and lower switching efficiency are resolved when mobile users are in an ultra-dense heterogeneous mobile network.

To resolve the problems of various time gaps in the network switching process in heterogeneous mobile networks, the A7 algorithm employed the motion trajectory data of the mobile terminal and machine learning model to predict and analyze the motion law of terminals and selected the best network switching access point from the prediction results to improve the timeliness and accuracy of the network switch. The problem could be solved by calculating the mobile terminal and the nearest network access point through the machine learning model to determine the best network switching access point. The common point with the A4 algorithm was that the monitored objects were all mobile end users. Although the four algorithms employed various key directions and methods to solve the problem, their software operation environment for the comparative experiment was the same. Therefore, the performance evaluation parameters with Experiments 1, 3, and 4 can be directly employed for comparative analysis. The outcomes of the experiments demonstrated the advantages of the presented algorithm.

## 5. Conclusion

The manuscript employed SDN technology to manage the resources of a mobile network system. The network switching mode is closely related to the strength of the signal of the APN access point. Besides, the game theory is introduced in the enhanced network handover method. Moreover, the transmission performance parameters of network evaluation, namely, throughput and transmission delay of the network, and packet loss rate of the MPTCP sub-flow transmission were introduced. The main conclusions of the research work were summarized as follows:

1. Through the integration and application of both SDN and MPTCP communication technologies, the mobile network communication model was constructed based on 5G and satellite communication technology. The application of network model technology involved a wide range of categories. The network communication model was mainly employed to collect the location or activity information of mobile phone users, realizing the running state management of the network and the transmission scheduling of the MPTCP sub-flow.
2. To detect the signal intensity information of the wireless network access of the mobile phone terminal continuously, the Kalman filter was utilized to calculate the best wireless network access point for mobile phone users.
3. Aiming at throughput, bandwidth consumption, and switching efficiency of the network, switching rules of the network were formulated, focusing on limiting the throughput of the sub-flow transmission path and the network bandwidth occupied by the sub-flow transmission path, and ensuring the speed of the network switching.
4. The delay parameter of the network was used to control the transmission performance, such as the sub-flow transmission rate, considering the influence of the network delay change and the network delay difference between the transmission paths on the transmission performance in sub-flow transmission.
5. By limiting the throughput of the sub-flow transmission path and the bandwidth occupied by sub-flow transmission, the network switching strategy was formulated to design and strengthen the ordinary transmission Moreover, the reliability, stability, and compatibility of the switching strategy were evaluated by using the Jacobian matrix.
6. With the signal strength of the APN access point, sub-flow transmission network bandwidth, and sub-flow transmission packet loss rate as main parameters, the performance evaluation strategy of network switching was developed. If all mobile phone terminals participated in the game and all APN access points were regarded as the goals of a game, the best APN access point was determined by calculating the balance point in potential game theory.

Future research will focus on the internal improvement of the MPTCP communication protocol, advance the transmission performance of MPTCP sub-streams and provide a stable and efficient transmission platform for the data stream. The research results of the 6G communication network can be combined with the current outcomes to improve the stability and reliability of the interface with satellite communication and more complex network connections and network switching can be ensured.

## Author contributions

All authors wrote and read the manuscript and agreed to the submission.
